# Mimicking Tissues in 3D-Printed Radiology Phantoms: Brand, Product, and Color of Printing Filaments Matter!

**DOI:** 10.3390/polym18070851

**Published:** 2026-03-31

**Authors:** Thomas Hofmann, Martin Buschmann, Adrian Belarra, Maria Castillo-Garcia, Margarita Chevalier, Irene Hernandez-Giron, Peter Homolka

**Affiliations:** 1 Center for Medical Physics and Biomedical Engineering, Medical University of Vienna, 1090 Vienna, Austria; 2 Division of Medical Radiation Physics, Department of Radiation Oncology, Medical University of Vienna, and University Hospital Vienna, 1090 Vienna, Austria; martin.buschmann@meduniwien.ac.at; 3 Radiology and Rehabilitation Department, Medical School, Complutense University of Madrid, 28040 Madrid, Spain; abelarra@ucm.es (A.B.); m.castillo.g@ucm.es (M.C.-G.); chevalier@med.ucm.es (M.C.); 4 School of Physics, University College Dublin, D04 V1W8 Dublin, Ireland; irene.hernandezgiron@ucd.ie

**Keywords:** radiographic phantoms, additive manufacturing, 3D printing, medical imaging, X-ray imaging, computed tomography, tissue-equivalent materials

## Abstract

Additive manufacturing enables the rapid fabrication of radiographic phantoms for X-ray and CT imaging, supporting applications such as patient simulation, dosimetry, imaging protocol optimization, and quality assurance. Polylactic acid (PLA) and acrylonitrile butadiene styrene (ABS) are among the most widely used printing polymers in phantoms; however, their X-ray attenuation properties can vary substantially among manufacturers, product lines within manufacturers, and even between colors of the same product. Cylindrical samples of 34 PLA filaments from 11 manufacturers and 13 ABS filaments from 9 manufacturers were evaluated for X-ray attenuation and energy dependence between 70 and 140 kV using a clinical CT scanner. Measured mass densities ranged from 1.17 to 1.34 g/cm^3^ for PLA and 1.03–1.11 g/cm^3^ for ABS. At 120 kV, Hounsfield unit (HU) values spanned 109 to 424 HU for PLA and −34 to 40 HU for ABS. Energy dependence, quantified as the HU at 70 kV minus HU at 140 kV, ranged from −29 to +172 HU for PLA filaments and −52 to −4 HU for ABS filaments. Identical products differing only in color showed HU variations from <2 HU to >90 HU at 120 kV, with no consistent pattern linking specific colors to highest or lowest attenuation. These findings demonstrate that 3D printing materials require individual characterization, as base polymer designation alone does not predict X-ray behavior accurately. The observed variability, however, enables the design of phantoms with tailored attenuation and energy-dependent contrast. Referring only to base polymers when specifying 3D printing materials for radiographic phantoms or suggesting printing materials as radiographic substitutes to mimic a specified tissue or reference material without naming the actual product, including color, is, thus, insufficient.

## 1. Introduction

Phantoms, physical or computational models that simulate study objects like the human body [1,2,3,4,5], replicating anatomical structures with varying degrees of realism, are widely used across all applications of ionizing radiation in medicine, from radiation therapy, diagnostic imaging, including radiology and nuclear medicine, to radiation protection and dosimetry. Phantoms need to mimic all relevant radiation interactions with the radiation field [6] with appropriate accuracy.

Their applications in diagnostic X-ray imaging include image and procedure optimization, virtual clinical trials, equipment testing, dosimetry and dose reconstruction, and quality assurance across all modalities such as computed tomography (CT), mammography, general radiography, as well as fluoroscopy and interventional imaging. Appropriate phantoms yield reproducible and reliable results without subjecting patients to radiation exposure. Although commercial phantoms offer established reproducibility for many, in most cases, simple routine tasks, their limitations and price point often require research groups to develop and build phantoms for a wide variety of applications, especially to target wider patient cohorts and anatomical and disease variability towards a more personalized and inclusive image quality evaluation and optimization [4].

Additive manufacturing technologies, mainly fused deposition modeling (FDM), stereolithography (SLA), and selective laser sintering (SLS), have had a significant impact on phantom research, enabling the rapid and cost-effective design and production of a wide variety of geometries.

For diagnostic medical X-ray imaging, the wide range of phantom applications covers applications using stylized to (pseudo)anthropomorphic phantoms [7] that incorporate anatomical noise textures [8,9], vascular hierarchies [10,11], lesion inserts [12], abdominal structures [13], commercial phantom insert alternatives [14], and modality-specific contrasts [12,15,16]. 3D printing offers an accessible and efficient approach to addressing the challenges associated with developing phantoms that accurately replicate anatomical structures [4,11,17,18], provided that suitable printing materials—primarily polymers—are used [19,20].

The exact chemical composition of 3D printing polymers is manufacturer proprietary and not disclosed to users [21,22]. Base polymer designation alone thus insufficiently predicts radiographic behavior or tissue equivalence, mandating product-specific characterization prior to phantom fabrication. Manufacturers frequently incorporate undisclosed ingredients and additives to improve printability, mechanical performance, or esthetic properties (e.g., colorants), resulting in products with distinct physical and mechanical characteristics or visual appearances [14,23] so nominally similar materials sharing the same polymer designation can exhibit different radiological properties. For example, “ABS+” typically denotes a vendor-modified ABS containing undisclosed additives designed to reduce shrinkage and warping and to improve bed adhesion compared with standard ABS [15]. Similarly, common commercial formulations of PLA and ABS are often modified to increase toughness (PLA) or to enable more accurate printing with reduced warping and lower emission of volatile organic compounds during printing (ABS) [24]. Different mineral (inorganic) and organic colorants are used to color the filaments, resulting in a priori unpredictable changes in X-ray attenuation properties. Actual measurements are therefore required for all colors of interest, even within the same filament series.

Consequently, numerous studies have focused on characterizing and cataloging the X-ray attenuation properties of commonly used 3D printing materials [15,18,22,25,26,27,28,29]. To broaden the range of achievable X-ray interaction properties or accurately mimic the attenuation behavior of specific materials or tissues, including bone, breast, and other soft tissues, or contrast agents, doping of these polymers with radiopaque additives is applied [30,31,32,33,34].

Filament-based FDM materials, particularly polylactic acid (PLA) and acrylonitrile-butadiene-styrene (ABS), exemplify materials well established for radiological phantoms within accessible printing ecosystems [15,33,35]. Among the materials most often suggested for 3D-printed phantoms, PLA, ABS, and HIPS are utilized to achieve a wide range of X-ray attenuation values from –1000 HU to above 500 HU [36]. Controlled underfilling of 3D printing materials can effectively replicate the X-ray attenuation properties of various tissues by reducing the mass density, enabling the production of accurate radiographic phantoms, and reducing the number of different polymers necessary to replicate various tissue densities [24].

Modern 3D printing polymers and filaments have undergone extensive optimization, yielding a diverse array of materials engineered for specialized performance characteristics. First-generation ABS filaments, for example, posed significant printing challenges due to thermal expansion and resultant shrinkage during post-extrusion solidification and cooling, often compromising bed adhesion by inducing warping. Contemporary formulations, however, facilitate reliable printing on consumer-grade equipment, even for users with modest expertise. Concurrent advancements in printer speeds have driven the development of high-throughput filaments that still maintain print quality. Specialized variants—offering enhanced mechanical properties or esthetic finishes for applications such as functional prototypes or decorative objects—were developed and replaced standard filaments. Notwithstanding these alterations, commercial filaments sharing the same base polymer designation (e.g., PLA or ABS) demonstrate considerable variability in X-ray attenuation. This arises from differences in additives, colorants, fillers, and polymer modifications, which modulate elemental composition and density, thereby influencing linear attenuation and its energy dependence. In X-ray imaging, the elemental composition is often summarized in the effective atomic number (Z_eff_), being a surrogate atomic number for multi-element mixtures or compounds, designed to characterize X-ray attenuation equivalent to that of a single-element material of atomic number Z_eff_ at a given X-ray energy or energy range [37,38,39], commonly used as a parameter quantifying tissue equivalence in radiographic or radiotherapy phantoms [40,41,42,43,44,45].

This study addresses these challenges by comparing common commercial PLA and ABS materials and quantifying the range of variation between different PLA and ABS variants or products, respectively, to facilitate their potential matching or classification as potential surrogates of relevant tissues or materials for anthropomorphic and tissue-equivalent phantoms in CT imaging.

## 2. Materials and Methods

### 2.1. Polymer Filaments Used in This Study

Standard commercially available printing filaments based on PLA and ABS were used in this study. In selecting the filaments, care was taken to include a wide range of currently available filaments optimized for various purposes, such as enhanced mechanical properties, ease or speed of printing, esthetic surface finish, and different colors. Filaments incorporating fibers, such as carbon, to further enhance mechanical properties were excluded. Due to the considerably larger variety of PLA-based filaments compared to ABS, a greater number of PLA than ABS filaments were investigated.

Table 1 lists the PLA filaments used, comprising 34 PLA-based filaments from 11 manufacturers. To explore how filament color affects X-ray attenuation within the same product line, one high-speed, easy-to-print PLA with improved mechanical properties (Elegoo Rapid PLA+, Shenzen Elegoo Technology Co., LTD, Shenzhen, China) was tested in five different colors. Transparent and matte filaments were also included, as transparency may be achieved through modification of the base polymer composition, blending or co-polymerization with more transparent polymers, while matte finishes may be obtained by adding mineral-based fillers increasing effective atomic number and attenuation. White filaments were included when multiple colors of the same filament type were selected, since a solid white color may be achieved by using mineral fillers like titanium dioxide, e.g.,

In Table 2 the ABS filaments used are listed. Since the influence of different filament colors can be expected to behave similarly to that observed for the PLA samples, a limited selection of colors was measured.

### 2.2. Sample Preparation and Evaluation

The printers used in this work were a Creality K2 Plus (Shenzhen Creality 3D Technology Co., Ltd., Shenzhen, China) and an Anycubic Kobra S1 (Shenzhen Anycubic Technology Co., Ltd., Shenzhen, China), both equipped with closed printing chambers, as well as an FLSUN V400 (Zhengzhou Chaokuo Electronic Technology Co., Ltd., Zhengzhou, China). Although the latter does not feature a closed temperature-controlled chamber, it provides direct visual monitoring of the printing process, facilitating identification and correction of printing issues and optimization of parameter settings.

Cylindrical samples with a diameter of 2 cm and a height of 3 cm were printed using nozzles with a 0.4 mm orifice and 0.15 mm layer height for all layers but the first (set to 0.2 mm for better bed adhesion). Printing speeds were adjusted according to the respective filament. The maximum speeds specified by the manufacturers, however, were reduced to ensure stable printing conditions. Particular attention was given to achieving the highest possible mass density of the printed cylinders in order to avoid or minimize air inclusions [22,46]. The printing parameters recommended by the manufacturers served as starting points for optimization for each filament type. Initially, flow rates were adjusted to the maximum feasible values for each printer using a standard PLA filament. The progress in printing technology and slicing software, combined with advances in material development compared to earlier studies [22,26], was clearly evident. All printers achieved optimal results in terms of maximum density—avoiding micro voids due to air inclusions and visible over-extrusion as effectively as possible—with a flow rate of 1.00 (100%) in PLA; however, two ABS filaments exhibited consistent over-extrusion at 100% flow rate, requiring reductions to 95.0% and 97.5%, respectively. Printing parameters for the individual filaments are detailed in Appendix A (Table A1 and Table A2, for PLA- and ABS-based filaments, respectively). These parameters were optimized for the printers employed and are thus partly printer-specific. Nevertheless, when using different printers, the stated values serve as a starting point. This holds particularly true for ambient and bed temperature settings when considering enclosed versus open-frame printers, as well as cooling settings specified as fan speeds (percent of maximum), which depend on each printer’s unique cooling architecture and airflow geometry. Note that chamber temperature is reported only for printers with an enclosed build chamber. For Fiberlogy HS PLA clear, printing speed was increased manually on the printer panel. However, a lower value when set in the slicing software would be recommended as a starting point. This material was printed in the open system (FLSUN V400) because printing failed in the Creality printer due to repeated breaking of the filament in the Teflon feeding tubes. A similar situation arose with 3DJake niceABS, which failed on the Creality printer due to bed adhesion issues and was therefore printed on the FLSUN system—despite ABS typically benefiting strongly from a closed, heated chamber. Automatic determination and adjustment of the flow rate using printer-implemented algorithms (available in two of the three printers used) resulted in slightly lower flow rates; therefore, this option was disabled.

The mass density of the samples was determined gravimetrically using a self-calibrating analytical laboratory balance (Sartorius AG, Göttingen, Germany) and measuring their dimensions using a digital caliper (Mitutoyo, Kawasaki, Japan). For each printed sample, three height measurements at different positions and six diameter measurements at different heights (top, middle, bottom) and perpendicular orientations were performed, and the corresponding averages were used in calculating volumes and mass densities.

To quantify the energy dependence of the polymer samples, the difference in HU values between 70 and 140 kV was calculated as

$$∆\mathrm{HU}\;=\;{\mathrm{HU}}_{70\mathrm{kV}}\;-\;{\mathrm{HU}}_{140\mathrm{kV}}$$

thus, negative values correspond to HU increasing with beam hardness, corresponding to materials with a lower effective atomic number than water, and positive values to HU decreasing for harder beams (effective atomic number higher than water). Percentage differences in CT numbers at 70 and 140 kV, expressed relative to their mean HU, were calculated as

∆HUHU + 1000 ∗ 100

shifting the mean value by 1000 to account for the correct zero attenuation level of the Hounsfield scale at −1000 HU.

### 2.3. CT Scanning of Samples

Samples were embedded in a cylindrical phantom of 20 cm diameter to minimize HU inaccuracies due to the CT scanner’s beam hardening correction [46]. The phantom consisted of 4 sections, each accommodating up to 17 samples arranged circularly at the center and radially in two concentric rings [46]. Four sample positions were reserved for PMMA tubes containing demineralized water to allow quantitative correction of the measured Hounsfield Unit (HU) values, ensuring a water value of exactly 0.00 HU and compensating for minor inaccuracies in CT calibration or beam hardening correction. The phantom sections were printed using Elegoo Rapid PLA+ with a 0.6 mm nozzle and 30% infill, applying a gyroid infill pattern to minimize potential imaging artifacts on the FLSUN V400.

Scanning was performed on a Siemens Somatom AS (Siemens Healthineers, Erlangen, Germany) applying a collimation width of 19.2 mm (32 × 0.6 mm) and a pitch factor of 0.55. A reconstructed slice width of 2 mm was used with a medium sharp kernel (B30s). For the 70 kV and 80 kV scans, the exposure was set to the maximum value corresponding to 900 and 1100 mAs, respectively, resulting in a CTDI_vol_ of 13.8 mGy (70 kV) and 27.05 mGy (80 kV). For the 100 kV, 120 kV, and 140 kV scans, the exposure was set to 1037 mAs, 1000 mAs, and 850 mAs, resulting in a CTDI_vol_ of 50 mGy for the 100 kV scan, 82.93 mGy (120 kV), and 101.43 mGy (140 kV), respectively. HU values in the samples were measured with cylindrical ROIs of 16 voxel radius corresponding to 14.4 mm radius, omitting the top and bottom slices to avoid partial volume artifacts, resulting in 12 HU measurements per sample from subsequent slices. HU values measured in the demineralized water in the same phantom section and radial distance in the phantom were subtracted to correct water HU values to exactly zero.

## 3. Results

**Figure 1:**
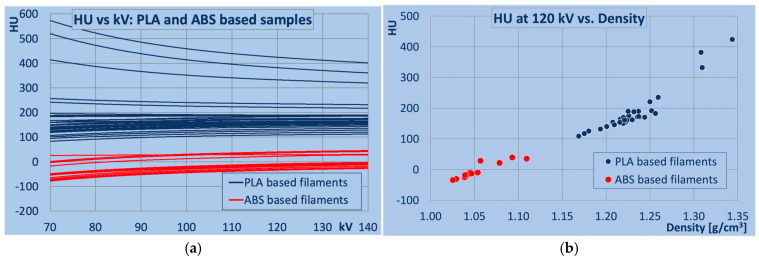
Overview of all PLA and ABS samples: (**a**) HU values measured versus tube potential (kV), (**b**) HU values at 120 kV versus mass density. Average measurement uncertainties in HU were below 2 HU, and for mass density, 0.006 g/cm^3^.

Figure 1 provides an overview of the results. Figure 1a shows the HU values and their energy dependence determined for all PLA- and ABS-based samples. In Figure 1b, the dependence of the HU values measured at 120 kV on the mass density of the printed samples is illustrated. 120 kV is used as a reference, since it represents the most common tube potential in CT scanning. Numeric values of HU at all kV and mass density of the samples are provided in Appendix A (Table A3 for PLA-, and Table A4 for ABS-based samples). In the PLA-based polymer samples, HU values at 120 kV ranged from 109 (Bambu PLA Tough+, cyan) to 424 (Polymaker Panchroma PLA Basic, cold white), with corresponding mass densities from 1.17 to 1.34 g/cm^3^. For ABS-based filaments, the minimum HU value at 120 kV was −34 (Fillamentum ABS Extrafill, natural), the maximum was 40 (Fiberlogy Easy ABS Pure, transparent), and mass densities ranged from 1.03 (Esun eABS+ HS, red) to 1.11 (3Djake niceABS, red).

### 3.1. PLA-Based Filaments

**Figure 2:**
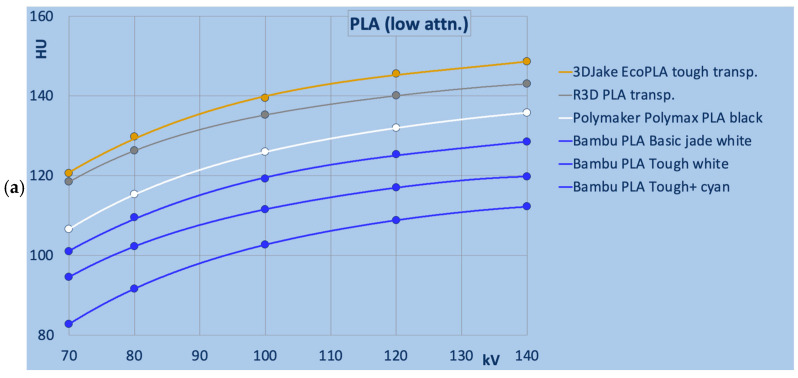

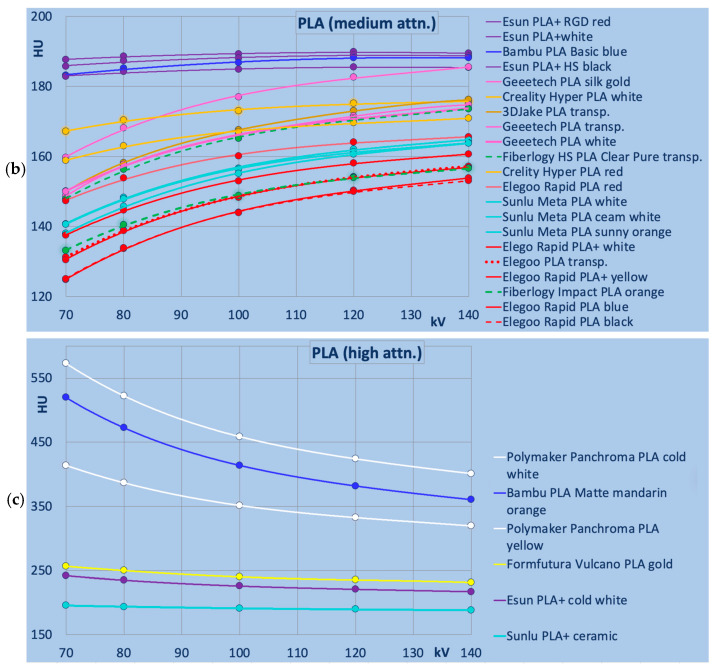
PLA-based samples in detail: (**a**) low attenuation; (**b**) medium attenuation; (**c**) higher attenuation. Note: in (**c**), HU values decrease with kV, whereas in (**a**,**b**) HU values increase with kV. Broken and dotted lines are used to allow discrimination of otherwise overlapping data. Note: colors indicate manufacturer.

**Figure 3:**
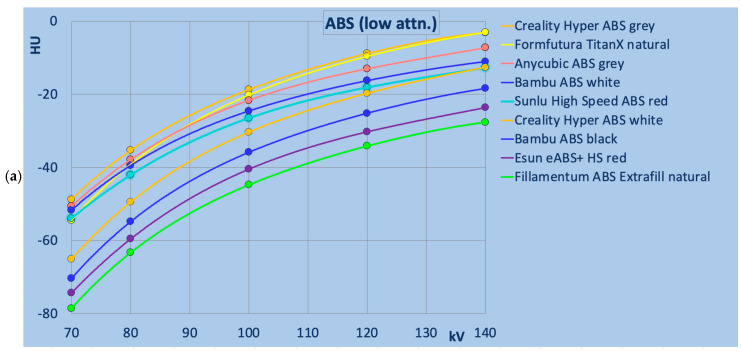

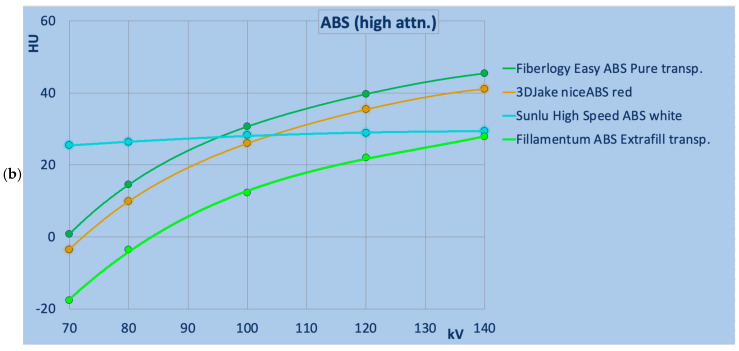
ABS samples in detail: (**a**) ABS with low attenuation (below 0 HU at 120 kV), and (**b**) higher attenuation (above 0 HU at 120 kV). Color coding indicating the manufacturer.

Figure 2 provides a detailed comparison of PLA-based filaments. In Figure 2 and Figure 3, different colors indicate different manufacturers. Figure legends are ordered from highest to lowest HU value at 120 kV. Figure 2a and b show PLA-based materials with the lowest and medium X-ray attenuations, respectively, where HU values increased with beam hardness from 70 to 140 kV. Most filaments with comparable attenuation exhibited similar energy dependence, though it was considerably larger in some in the medium attenuation group, as in Geeetech PLA silk gold, 3DJake PLA transparent, and Geeetech PLA transparent and white. In these filaments, the relative energy dependence amounted to 2.0 to 2.2% (in absolute values, −24 to −26 HU), compared to −0.2% to −0.7% (absolute −3 to −12 HU) in the Creality Hyper PLAs (shown in orange) and Esun PLA + HS (purple in the figure), exhibiting the closest attenuation at 120 kV.

The PLA filaments exhibiting the highest X-ray attenuation (cf. Figure 2c) all showed decreasing HU values with increasing beam hardness.

### 3.2. ABS-Based Filaments

Figure 3 presents detailed data for ABS-based printing polymers. Low- and high-attenuation materials were separated at a reference value of 0 HU at 120 kV. All ABS filaments exhibited increasing HU values with tube voltage (70–140 kV), Sunlu High-Speed ABS white (in Figure 3b) exhibited a minimal energy dependence, with a difference of −4 HU between 70 and 140 kV (−0.4%). All other ABS samples displayed a more pronounced energy dependence, with HU differences ranging from −41 to −52 HU (−4.3% to −5.5%).

### 3.3. Effect of Colorants Within Filament Types

**Figure 4:**
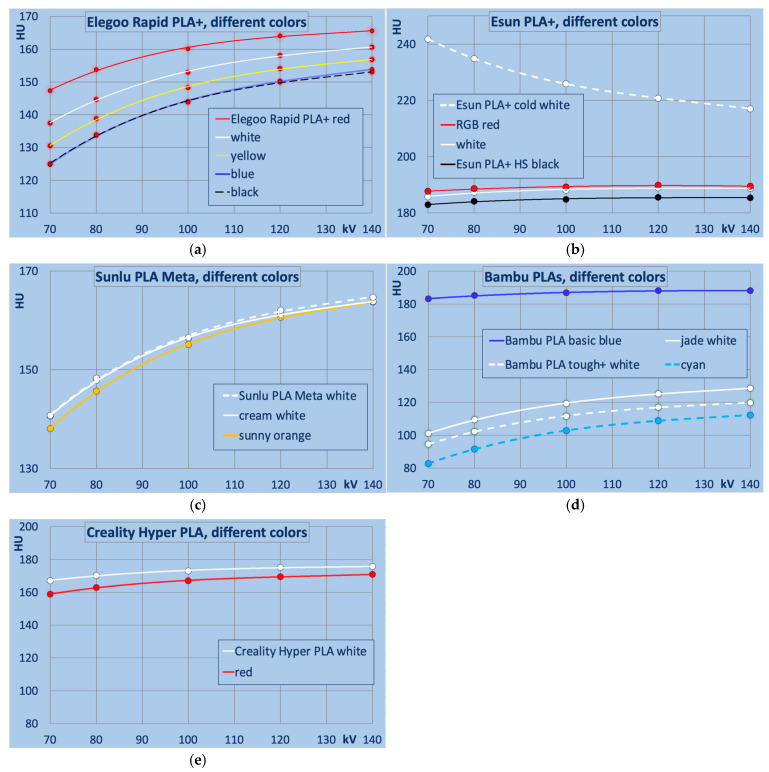
Differences between PLA filament by colors. (**a**) Elegoo Rapid PLA+, (**b**) Esun PLA+, (**c**) Sunlu PLA Meta, (**d**) Bambu (solid lines: PLA basic, dashed lines: PLA Tough+), and (**e**) Creality PLA-based filaments.

**Figure 5:**
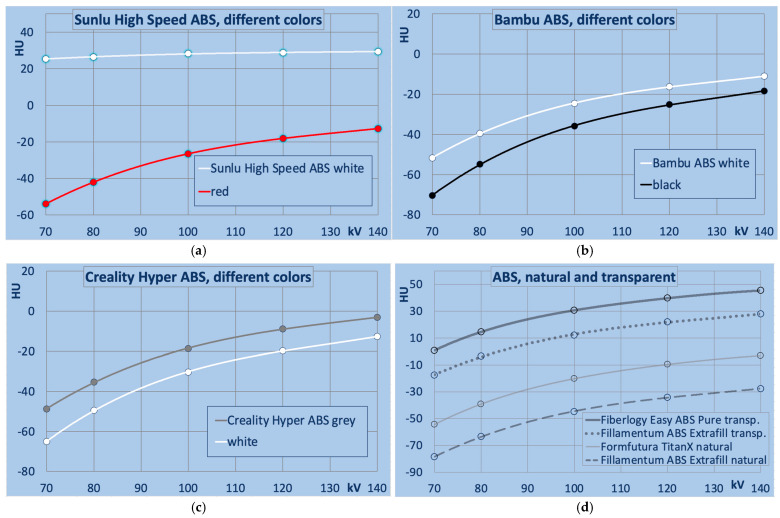
Differences between colors for ABS filaments. (**a**) Sunlu high-speed ABS; (**b**) Bambu ABS; (**c**) Creality Hyper ABS; and (**d**) natural and transparent ABS filaments.

A distinct dependence of X-ray attenuation on filament color is observed in certain filament types. Figure 4 and Figure 5a–c compare filaments that differ exclusively in color. For ease of interpretation, the colors in these figures correspond to those of the respective filaments.

Figure 4 shows differences between filament colors within the same PLA filament type. While the variation in some filaments is minimal between colors (as in Sunlu PLA Meta for the colors tested with <3HU between Sunlu Meta white and orange at 120 HU), it is approximately 34 HU in Esun PLA+ cold white vs. black, and exceeds 60 HU in Bambu PLA Basic white vs. blue.

Figure 5a–c illustrate the effects of different color pigments in ABS-based filaments. The largest deviation is found in Sunlu High Speed ABS. The white variant exhibits a flatter energy dependence in combination with a higher attenuation (79 to 42 HU difference at 70 and 140 kV, respectively, and 47 HU higher at the reference tube potential of 120 kV). In Bambu ABS and Creality Hyper ABS HU differences of ~10 HU at 120 kV are seen.

Figure 5d illustrates HU values of transparent and natural ABS variants. Fillamentum ABS Extrafill natural exhibited the lowest attenuation among all ABS filaments; transparent variants showed higher attenuation and slightly higher mass density than natural ABS and all other ABS tested, except for 3DJake niceABS. Filaments marketed as ABS natural and ABS transparent, despite not containing pigments, still vary considerably in their X-ray attenuation. It should be noted that pure ABS is not transparent but rather slightly opaque, so the transparent variants most likely consist of copolymers or otherwise modified base polymers, despite the filaments’ names.

**Figure 6:**
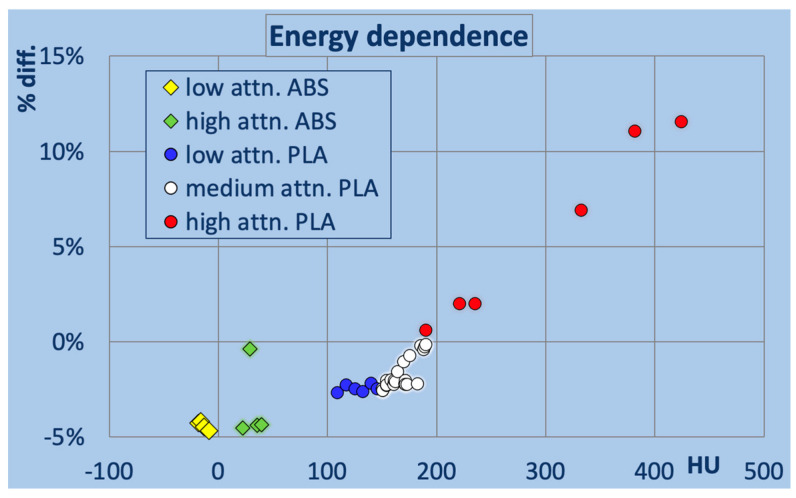
Percentage energy dependence of HU between 70 vs. 140 kV.

The printed samples exhibit distinct relative energy dependencies across the tested range (70–140 kV in CT). Figure 6 shows the percentage difference in attenuation for the softest and hardest spectrum used relative to the mean value as a function of the HU value at 120 kV. All ABS and the low- and medium-attenuation PLA exhibit higher attenuation at harder beams, as seen in a negative value. Most ABS-based filaments show deviations close to −5% (−5.5% to −4.4%), except for Sunlu High Speed ABS (white) at −0.4%. Low-attenuation PLAs range from −2.7% to −2.2%, medium-attenuation PLAs from −2.5% to −0.2%, while high-attenuation PLA-based polymers demonstrate approximately linear increases up to >10% with rising HU values, as expected from higher effective atomic number fillers.

## 4. Discussion

The imperfect correlation between mass densities and HU values indicates variations in effective atomic number among filaments using the same base polymer depending on filament series and color. This suggests that compositional differences, rather than density alone, influence X-ray attenuation in the investigated materials. From an application perspective, when using 3D printing for X-ray phantom fabrication, the wide variation in both absolute attenuation and the energy dependence of attenuation leads to three important practical implications:

First, each individual 3D printing material must be characterized across the relevant X-ray energy range in which the phantom will be used (considering kV and filtration to account for CT manufacturers and variability in protocols), to verify its suitability for the intended application.

Second, the wide range of X-ray attenuation in PLA- and ABS-based filaments—with PLA generally covering positive HU values and ABS extending to negative ones—enables simulation of a broad spectrum of tissues and contrasts using only these two base materials. This range can be further extended by applying controlled underfilling, adjusting the filament extrusion rate to reduce and tailor X-ray attenuation by typically a maximum of 10–20% [24,47]. However, because density reduction achieved by underfilling compromises structural stability if excessive [47,48], it should be kept minimal to moderate, and alternative filaments—most likely different colors or filament series—should be selected instead when adjustments exceeding a few percent are required [22,46,49]. This also holds true for density reduction via (typically periodic) infill patterns, with reduced infill density representing an alternative approach. However, infill patterns are used for density reduction [28,45,50,51,52] and likely introduce inhomogeneity patterns, increased noise, altered noise power spectra, and partial volume effects that differ from typical anatomical noise—all generally undesirable effects that can be avoided or greatly reduced by appropriate material selection from the wide range of finely graduated X-ray attenuation properties available.

Third, one must be aware that ongoing product optimization may lead to new batches or revised versions of existing filaments with altered X-ray attenuation properties, and that published phantom designs specifying only the base polymer (without exact filament products and colors) or relying on filaments that are no longer available offer limited reproducibility.

In low- and medium-attenuation PLA materials, HU values increased with harder radiation, consistent with typical base polymer behavior. In contrast, all high-attenuation PLA filaments exhibited decreasing HU values with increasing beam hardness, indicating mineral fillers or pigments that increase their effective atomic number above that of water. The top three—optimized for esthetic/matte appearance—likely contain such fillers: Polymaker Panchroma PLA Basic cold white, Bambu PLA Matte mandarin orange, and Polymaker Panchroma PLA Basic yellow. FormFutura Volcano PLA in gold color, a technical high-temperature, post-curable filament, appears next in the attenuation ranking, followed by eSun PLA+ cold white and Sunlu PLA+ ceramic. Note that the “ceramic” designation in Sunlu PLA+ refers only to its color and matte appearance, not to actual ceramic content. This behavior in phantom materials mirrors that observed in human tissues and clinical X-ray contrasts: higher HU values above regular soft tissue are associated with elevated effective atomic numbers, causing HU values to decrease with increasing beam energy.

Unlike high-attenuation PLA filaments, all ABS materials showed increasing HU values with beam hardness, consistent with typical polymer behavior lacking heavy fillers.

Elegoo Rapid PLA+ showed substantial color-to-color variability, with red exhibiting the highest attenuation, followed by white and yellow to blue/black with the lowest and nearly identical attenuation. Esun PLA+ “cold white” displayed substantially higher attenuation and decreasing HU with beam hardness, unlike standard “white” (intermediate between red and black). Sunlu PLA Meta exhibited minimal color differences overall. Creality Hyper PLA reversed the trend (white > red). Bambu PLA Tough+ white showed high attenuation similar to Elegoo; however, PLA Basic blue, relative to “jade white”, exhibited the largest difference related to color pigments among all tested filament series (ΔHU: 82 at 70 kV, 60 at 140 kV).

In Sunlu High-Speed ABS white, elevated white pigment content associated with a higher effective atomic number produced flatter energy dependence and increased attenuation. The minimal energy dependence, seen as almost constant attenuation relative to water and thus, a relative kV-independent HU value, across diagnostic energies in the range used in CT, is notable. It may be considered, thus, a preferred choice for an almost water-equivalent phantom material in CT. To reduce HU slightly from 29 HU (at 120 kV) to about 0, a minimal density reduction by adjusting the flow rate in the printing process accordingly [24], can be applied, however.

Bambu and Creality ABS showed comparable energy dependence to red Sunlu ABS. Greater inter-brand variability appeared in transparent ABS variants. Fillamentum natural (unpigmented) ABS displayed the lowest attenuation overall in the ABS-based filaments tested, attributable to the absence of colorants, while transparent variants’ higher attenuation and mass density suggest alterations in the base polymer. Due to the negative HU values and appropriate energy dependence of pure ABS, several ABS-based filaments represent suitable surrogates for adipose tissues in radiographic phantoms.

The results of this work align with the preceding studies on X-ray attenuation of 3D printing materials [22,26,46] regarding PLA and ABS. The too-low effective atomic number seen in the energy dependence of HU values is still found in many of the PLA and ABS materials, especially in those with lower overall attenuation. Notably, while those studies observed some variations in FDM filaments sharing the same base polymers, this investigation demonstrates that polymers with the same base designation (PLA, ABS) and even manufacturer and product line can differ markedly in their radiographic attenuation properties. In the 2021 study, the diversity of available PLA and ABS materials was substantially lower, and these materials were more challenging to print (particularly ABS) or required slower printing speeds (PLA and ABS). The variations in X-ray attenuation properties and energy dependence demonstrated in this study can further be exploited to achieve targeted radiation contrasts in phantoms through material selection, where not only product line and manufacturer but also color—owing to incorporated pigments and colorants—play critical roles. This substantially expands the datasets and thus the possibilities outlined in the two preceding studies [22,46].

The 3D-printed materials identified as promising for CT tissue matching warrant benchmarking against patient data and commercial phantoms of well-defined material composition. Further studies are needed to evaluate the consistency and reproducibility of these candidate materials. The results found in terms of material attenuation (HU values) were obtained on solid samples with a simple cylindrical shape and may differ when more complex anatomical structures are printed. The samples were scanned in a specific CT system under one protocol for a range of kV; however, a multivendor study has not been performed. The characterization of the homogeneity (or lack thereof) of the 3D-printed materials was out of the scope of this study, but can be of interest when considering realistic anatomical structures, such as ground glass nodules and other tissues/pathologies, where this has implications in patient diagnosis.

## 5. Conclusions

3D printing materials sharing the same base polymer designation (PLA and ABS) exhibit wide variations in composition, leading to substantial differences in X-ray attenuation properties. Consequently, both the specific product and color selected for printing radiographic phantoms significantly influence X-ray attenuation. Manufacturers’ ongoing product optimizations further necessitate vigilant monitoring of compositional changes. On the positive side, this variability enables the creation of a broad spectrum of finely graduated contrasts and tissue-mimicking attenuations using a limited selection of base polymers like PLA and ABS, where ABS provides lower attenuation suitable for adipose tissue equivalents, and PLA based filaments achieve up to 400+ HU mimicking more X-ray opaque tissues.

## Figures and Tables

**Table 1:** PLA filaments used (34 filaments from 11 manufacturers).

Manufacturer	Filament	Color(s)
3DJake (Niceshops GmbH, Paldau, Austria)		
	PLA	transparent
	EcoPLA Tough	transparent
Bambu (Bambulab Ltd., Kowloon, Hong Kong, China)		
	PLA Basic	blue, jade white
	PLA Matte	Mandarin orange
	PLA Tough+	white, cyan
Creality (Shenzhen Creality 3D Technology Co, Ltd., Shenzhen, China)		
	HyperPLA	white, red
Elegoo (Shenzen Elegoo Technology Co., LTD, Shenzhen, China)		
	Rapid PLA+	red, white, yellow, blue, black
	PLA Translucent	transparent
	PLA Pro	light blue
Esun (Shenzhen Esun Industrial Co., Ltd., Shenzhen, China)		
	PLA+	cold white, RGB red, white
	PLA+ HS	black
Fiberlogy (Fiberlogy SA, Brzezie, Poland)		
	HS PLA Clear	pure transparent
	Impact PLA	orange
Form Futua (Formfutura BV, Nijmegen, The Netherlands)		
	Volcano PLA	gold
Geeetech (Shenzhen Getech Technology Co., Ltd., Shenzen, China)		
	PLA	silk gold, transparent, white
Polymaker (JF Polymers Co., Ltd., Changshu, China)		
	Panchroma PLA Basic	cold white, yellow
	Polymax PLA	black
R3D (Wuhu R3D Technology Co., Ltd., Wuhu, China)		
	PLA	transparent
Sunlu (Zhuhai Sunlu Industrial Co., Ltd., Zhongshan, China)		
	PLA+	ceramic (white)
	PLA Meta	white, cream white, sunny orange

**Table 2:** ABS filaments used (13 filaments from 9 manufacturers).

Manufacturer	Filament	Color(s)
3DJake (Niceshops GmbH, Paldau, Austria)		
	niceABS	red
Anycubic (Shenzhen Anycubic Technology Co., Ltd., Shenzhen, China)		
	ABS (RFID)	gray
Bambu (Bambulab Ltd., Kowloon, Hong Kong, China)		
	ABS	white, black
Creality (Shenzhen Creality 3D Technology Co, Ltd., Shenzhen, China)		
	Hyper ABS	gray, white
Esun (Shenzhen Esun Industrial Co., Ltd., Shenzhen, China)		
	eABS+ HS	red
Fiberlogy (Fiberlogy SA, Brzezie, Poland)		
	Easy ABS	pure transparent
Fillamentum (Fillamentum Manufacturing Czech s.r.o., Hulín, Czech Republic)		
	ABS Extrafill	transparent, natural
Form Futua (Formfutura BV, Nijmegen, The Netherlands)		
	TitanX	natural
Sunlu (Zhuhai Sunlu Industrial Co., Ltd., Zhongshan, Guangdong, China)		
	High-Speed ABS	white, red

## Data Availability

The original contributions presented in this study are included in the article. Further inquiries can be directed to the corresponding author.

## Abbreviations

The following abbreviations are used in this manuscript:

ABS	Acrylonitrile butadiene styrene
CT	Computed tomography
FDM	Fused deposition modeling
HIPS	High-Impact Polystyrene
HU	Hounsfield Unit
PLA	Polylactic acid
SLA	Stereolithography Apparatus

## Appendix A

Printing parameters and identification of the printer used for each individual sample are provided in Table A1 (PLA) and Table A2 (ABS). HU values and mass densities determined for polymer samples are shown in Table A3 and Table A4. Average measurement uncertainties derived from slice-to-slice variation in HU values were below 2 HU in the printed polymer samples for all kV values. Measurement uncertainties for mass density were ≤0.006 g/cm^3^.

**Table A1:** Printers and printing parameters used for PLA samples.

Manufacturer	Filament Name	Printer Used	Temperatures (Nozzle/Bed/Chamber) [°C]	Print Speed (Infill/Walls) [mm/s]	Cooling Fan Speed	Flow Rate
Elegoo	Rapid PLA+	FLSUN V400	220/60	100/100	100%	1
	PLA Translucent	FLSUN V400	220/60	60/60	100%	1
	PLA PRO light blue	Creality K2+	205/50/35	90/60	50%	1
Creality	HyperPLA	Creality K2+	220/50/35	90/90	100%	1
Form Futura	Volcano PLA	Creality K2+	250/50/35	90/60	100%	1
Fiberlogy	HS PLA Clear	FLSUN V400	230/60	240/240 *	100%	1
	Impact PLA	Creality K2+	235/50/35	90/60	100%	1
Esun	PLA+	FLSUN V400	220/60	100/100	100%	1
	PLA+ HS	Creality K2+	230/50/35	100/100	100%	1
Sunlu	PLA+	FLSUN V400	200/60	90/90	100%	1
	PLA Meta	FLSUN V400	220/60	90/90	100%	1
Bambu	PLA Basic	Creality K2+	210/50/35	90/90	100%	1
	PLA Matte	Creality K2+	210/50/35	90/90	100%	1
	PLA Tough+	Creality K2+	235/50/35	90/90	100%	1
Polymaker	Panchroma PLA Basic	FLSUN V400	210/60	100/100	100%	1
	Polymax PLA	Creality K2+	220/50/35	90/90	100%	1
Geeetech	PLA	Creality K2+	210/50/35	90/90	100%	1
R3D	PLA	Creality K2+	210/50/35	90/90	100%	1
3Djake	PLA	Creality K2+	210/50/35	90/90	100%	1
	EcoPLA Tough	FLSUN V400	210/60	90/90	100%	1

* controlled manually during print.

**Table A2:** Printers and printing parameters used for ABS samples.

Manufacturer	Filament Name	Printer Used	Temperatures (Nozzle/Bed/Chamber) [°C]	Print Speed (Infill/Walls) [mm/s]	Cooling Fan Speed	Flow Rate
Esun	eABS+ HS	Creality K2+	260/100/50	100/100	10%	1
Formfutura	TitanX	Creality K2+	260/100/50	50/50	10%	1
Fiberlogy	Easy ABS	Creality K2+	245/100/60	60/50	30%	1
Creality	Hyper ABS	Creality K2+	260/80/50	90/60	10%	1
3DJake	niceABS	FLSUN V400	240/90	60/30	50%	1
Fillamentum	ABS Extrafill	Creality K2+	230/90/50	50/50	25%	1
Bambu	ABS	Anycubic Kobra S1	260/80/50	90/60	10%	1
Sunlu	High-Speed ABS	Anycubic Kobra S1	270/100/-	100/100	80%	0.95
Anycubic	ABS (RFID)	Anycubic Kobra S1	270/100/-	100/100	80%	0.975

**Table A3:** HU values and mass density determined for PLA-based polymer printed samples.

Manufacturer	Filament Name	Color	HU 70 kV	HU 80 kV	HU 100 kV	HU 120 kV	HU 140 kV	Density (g/cm^3^)
Elegoo	Rapid PLA+	red	147.3	153.8	160.2	164.1	165.6	1.22
	Rapid PLA+	white	137.5	144.7	153.0	158.1	160.6	1.22
	Rapid PLA+	yellow	130.5	138.8	148.3	154.1	156.8	1.22
	Rapid PLA+	blue	124.8	133.7	144.0	150.3	153.8	1.22
	Rapid PLA+	black	125.0	133.9	143.9	150.0	153.0	1.22
	PLA Translucent	transparent	131.2	138.9	148.5	154.1	157.1	1.21
	PLA PRO light blue	light blue	124.8	133.7	144.0	150.3	153.8	1.22
Creality	HyperPLA	white	167.1	170.4	173.0	175.1	175.7	1.23
	HyperPLA	red	158.8	162.9	167.0	169.5	170.9	1.22
Form Futura	Volcano PLA	gold	256.4	249.8	240.0	235.3	231.4	1.26
Fiberlogy	HS PLA Clear	pure transparent	147.8	156.2	165.1	170.6	173.5	1.24
	Impact PLA	orange	133.2	140.5	148.8	154.0	156.5	1.22
Esun	PLA+	cold white	241.8	234.8	225.9	220.8	217.0	1.25
	PLA+	RGB red	187.7	188.6	189.2	189.8	189.5	1.24
	PLA+	white	185.7	187.3	188.1	188.9	188.7	1.23
	PLA+ HS	black	182.9	184.2	184.8	185.5	185.4	1.23
Sunlu	PLA+	ceramic	195.7	193.6	190.9	189.8	188.3	1.23
	PLA Meta	white	140.7	148.3	156.6	162.1	164.7	1.23
	PLA Meta	cream white	140.6	147.9	156.3	161.3	163.8	1.22
	PLA Meta	sunny orange	138.1	145.7	155.1	160.6	163.7	1.22
Bambu	PLA Basic	blue	183.2	185.2	186.8	188.1	188.1	1.23
	PLA Basic	jade white	101.0	109.5	119.2	125.3	128.5	1.18
	PLA Matte	mandarin orange	519.6	472.5	413.7	381.7	360.4	1.31
	PLA Tough+	white	94.6	102.3	111.6	117.0	119.8	1.18
	PLA Tough+	cyan	82.8	91.6	102.7	108.8	112.2	1.17
Polymaker	Panchroma PLA Basic	cold white	572.9	522.2	458.7	424.1	401.1	1.34
	Panchroma PLA Basic	yellow	413.9	386.6	351.4	332.4	319.4	1.31
	Polymax PLA	black	106.6	115.3	125.9	132.0	135.8	1.19
Geeetech	PLA	silk gold	159.7	168.1	176.9	182.6	185.5	1.26
	PLA	transparent	148.7	157.2	166.3	171.5	174.7	1.24
	PLA	white	150.0	157.6	165.8	171.0	173.7	1.24
R3D	PLA	transparent	118.4	126.3	135.2	140.1	143.0	1.20
3Djake	PLA	transparent	150.0	158.2	167.6	173.0	176.2	1.24
	EcoPLA Tough	transparent	120.6	129.7	139.5	145.5	148.6	1.21

**Table A4:** HU values and mass density determined for ABS-based polymer printed samples.

Manufacturer	Filament Name	Color	HU 70 kV	HU 80 kV	HU 100 kV	HU 120 kV	HU 140 kV	Density (g/cm^3^)
Esun	eABS+ HS	red	−74.4	−59.6	−40.5	−30.3	−23.6	1.03
Formfutura	TitanX	natural	−54.4	−39.2	−20.2	−9.6	−3.1	1.05
Fiberlogy	Easy ABS	pure transparent	0.8	14.5	30.7	39.7	45.4	1.09
Creality	Hyper ABS	gray	−48.8	−35.3	−18.7	−8.8	−3.1	1.05
	Hyper ABS	white	−65.0	−49.5	−30.3	−19.7	−12.6	1.04
3DJake	niceABS	red	−3.5	9.9	26.0	35.4	41.1	1.11
Fillamentum	ABS Extrafill	transparent	−17.6	−3.6	12.2	22.0	27.9	1.08
	ABS Extrafill	natural	−78.6	−63.3	−44.8	−34.2	−27.6	1.03
Bambu	ABS	white	−51.7	−39.5	−24.5	−16.3	−11.1	1.04
	ABS	black	−70.4	−54.8	−35.9	−25.2	−18.4	1.04
Sunlu	High-Speed ABS	white	25.4	26.4	28.2	28.8	29.4	1.06
	High-Speed ABS	red	−53.9	−42.1	−26.5	−18.1	−12.7	1.04
Anycubic	ABS (RFID)	gray	−50.6	−37.9	−21.6	−13.0	−7.3	1.05
